# Transcutaneous Levator Aponeurosis Recession Without Spacer Grafts for Upper Eyelid Retraction: A Five-Case Series

**DOI:** 10.7759/cureus.112401

**Published:** 2026-07-10

**Authors:** Tatsuya Sakamoto, Ayano Yoshimura, Yoshikazu Kanemori

**Affiliations:** 1 Ophthalmology, Tsukazaki Hospital, Himeji, JPN; 2 Ophthalmology, Nagoya City University Hospital, Nagoya, JPN; 3 Ophthalmology, Hyogo Medical University, Nishinomiya, JPN; 4 Ophthalmology, Kanemori Eye Plastic Surgery Clinic, Kobe, JPN

**Keywords:** imagej, levator aponeurosis recession, oculoplastic surgery, thyroid eye disease, upper eyelid retraction

## Abstract

This case series describes the surgical technique and early postoperative outcomes of transcutaneous levator aponeurosis recession without spacer grafts or newly created flaps for upper eyelid retraction. We report five consecutive Japanese patients (five eyelids) who underwent this procedure at Kanemori Eye Plastic Surgery Clinic between July 2017 and February 2026. Preoperative and three-month postoperative images were analyzed using ImageJ (National Institutes of Health; Bethesda, Maryland, United States). Palpebral fissure height ratio (PFHR) and upper eyelid margin height ratio (UEMHR) were calculated by normalizing palpebral fissure height and upper eyelid margin height to the white-to-white distance (WTW). Each image was measured three times, and the mean value was used for descriptive analysis. The median PFHR of the operated eyelid decreased from 111.18% (range, 63.57-132.16%) preoperatively to 89.93% (range, 59.50-99.71%) postoperatively. The median UEMHR decreased from 57.59% (range, 28.20-77.38%) to 37.72% (range, 25.17-50.09%). PFHR and UEMHR decreased in all five operated eyelids, and intereye asymmetry in both indices decreased. Exposure-related superficial punctate keratopathy in Cases 2, 3, and 5 and lagophthalmos in Case 4 resolved postoperatively. During the three-month follow-up, no infection, hematoma, diplopia, new severe lagophthalmos, or overcorrection/undercorrection requiring reoperation was observed. These early findings suggest that this spacer-free technique is feasible in selected cases and may provide consistent eyelid lowering when assessed with WTW-normalized eyelid height indices; however, these non-standard photographic indices are not substitutes for prospectively measured margin reflex distance 1 (MRD1). Larger prospective studies with longer follow-up, standardized MRD1 and ocular surface assessment, masked independent graders, and formal measurement reproducibility analysis are needed to assess durability, recurrence, late eyelid contour changes, measurement reliability, and etiology-specific suitability.

## Introduction

Upper eyelid retraction can occur after eyelid surgery, trauma, inflammation, or thyroid eye disease (TED) [1,2]. TED-related upper eyelid retraction may present as lid retraction or lid lag and may coexist with proptosis or extraocular muscle involvement [1,2]. Excessive upper eyelid elevation can cause cosmetic concerns, a staring appearance, lagophthalmos, foreign body sensation, exposure keratopathy, and punctate epithelial erosions [1,2]. When conservative treatment is insufficient, upper eyelid lengthening may be required.

For readers less familiar with oculoplastic surgery, the levator aponeurosis is the distal tendon-like portion of the main upper eyelid elevator that inserts onto the tarsus, whereas the Mueller muscle is a posterior smooth-muscle retractor that also contributes to upper eyelid position. Upper eyelid lengthening procedures aim to reduce excessive eyelid elevation by weakening, releasing, recessing, or lengthening these retractors. Spacer grafts or flaps are used in some techniques to maintain eyelid lengthening, but they may require additional tissue handling, flap preparation, or graft-related considerations.

Several operations have been described for upper eyelid retraction, including graded full-thickness anterior blepharotomy [3], modified marginal myotomy [4], levator recession with adjustable sutures [5], levator lengthening using an orbital septal flap [6-8], V-Y levator lengthening [9], and stepwise management for iatrogenic upper eyelid retraction [10]. Although these methods can be useful, they may require full-thickness eyelid incision, adjustable sutures, spacer grafts, orbital septal flap preparation, or V-Y plasty [3-10]. Therefore, a transcutaneous approach that avoids spacer grafts and newly created flaps may be relevant for selected patients when adequate release of the levator aponeurosis-Mueller muscle complex can be achieved.

We have used a transcutaneous approach similar to that used to expose the levator aponeurosis in standard ptosis surgery. Instead of advancing the levator aponeurosis, however, we recess the levator aponeurosis-Mueller muscle complex and reattach only the distal end of the levator aponeurosis to the tarsus. This case series describes the surgical technique and early postoperative outcomes in five consecutive patients with upper eyelid retraction of different etiologies.

## Case presentation

Case series design and ethical considerations

This retrospective case series included patients who underwent transcutaneous levator aponeurosis recession for upper eyelid retraction at Kanemori Eye Plastic Surgery Clinic. Five consecutive patients (five operated eyelids) treated between July 2017 and February 2026 were included. Indications included upper eyelid retraction after prior eyelid surgery and TED-related or suspected inactive TED-related upper eyelid retraction. Patients with severe ocular surface disease unrelated to upper eyelid retraction that would preclude postoperative evaluation were excluded. Exposure-related keratopathy caused by upper eyelid retraction was not an exclusion criterion. Patients with a history of orbital surgery were excluded.

This retrospective case series was determined by Kanemori Eye Plastic Surgery Clinic, in accordance with its institutional policy, to be exempt from formal institutional review. The study adhered to the tenets of the Declaration of Helsinki. The manuscript does not include patient names, initials, medical record numbers, dates of birth, exact visit dates, or other direct identifiers. For the patients whose clinical images are included in the published manuscript, written informed consent was obtained for open-access publication of preoperative and postoperative facial/periocular images, including potentially identifiable images. The signed consent forms specifically acknowledged that the images may disclose privacy and personally identifiable information and may allow patient recognition despite efforts to remove or obscure direct identifiers. Case 4 is presented only as de-identified clinical information in the text and tables, and no clinical images of Case 4 are included because signed written documentation authorizing publication of potentially identifiable images was not available for editorial verification.

Photographic assessment

Preoperative and postoperative frontal-view images were used for image analysis. Images were obtained with the patient in primary gaze and natural eyelid opening, with the frontalis muscle relaxed as much as possible. Images with obvious head tilt, incomplete blinking, forced excessive eyelid opening, or gaze deviation were excluded from measurement. Postoperative evaluation was performed at approximately three months after surgery in all patients. In Case 2, postoperative image analysis was performed using the final postoperative appearance after additional left upper eyelid levator advancement.

Image analysis was performed using ImageJ (National Institutes of Health; Bethesda, Maryland, United States) [11]. In each image, the horizontal corneal diameter was defined as the white-to-white distance (WTW). Palpebral fissure height (PFH) was defined as the distance from the upper eyelid margin to the lower eyelid margin along a vertical line passing through the corneal center. Upper eyelid margin height (UEMH) was defined as the vertical distance from the corneal center to the upper eyelid margin and was recorded as a positive value when the upper eyelid margin was located above the corneal center.

Because absolute distances in millimeters could not be calculated reliably from the images, WTW was used as an internal reference. For each image, three measurement sets were performed. In each measurement set, the WTW was reidentified and used to calibrate the image scale to 10 arbitrary units in ImageJ. PFH and UEMH were then measured under the same calibration. In each set, PFH and UEMH were divided by the WTW measured in the same set and multiplied by 100 to calculate the palpebral fissure height ratio (PFHR) and upper eyelid margin height ratio (UEMHR), respectively: PFHR (%) = PFH/WTW x 100; UEMHR (%) = UEMH/WTW x 100. Thus, PFHR and UEMHR represented WTW-normalized relative indices rather than absolute millimeter measurements. The mean values of the three PFHR and UEMHR measurements were used as the representative values for analysis. Repeated measurements were performed to reduce measurement variability; the independent statistical unit was the patient. All image measurements were performed by a single observer. The observer was not masked to the preoperative or postoperative status because of the retrospective nature of the study.

The standard deviations of triplicate measurements were used only to describe within-image repeatability by the same observer and were not intended to represent formal intraobserver or interobserver measurement reliability.

Margin reflex distance 1 (MRD1) was not recorded prospectively in a standardized manner. Retrospective MRD1 assessment was considered because a corneal light reflex was visible in some images; however, the images were not obtained with a millimeter scale. Therefore, any retrospective MRD1 value would have required WTW-based calibration and would have shared the same photographic and WTW-dependent limitations as the primary indices. To avoid presenting potentially misleading millimeter estimates, MRD1 was not reported.

Surgical procedure

Subcutaneous infiltration anesthesia was performed with approximately 1.5-2.0 mL of 2% lidocaine with epinephrine. The upper eyelid skin incision was marked approximately 5-7 mm above the eyelid margin. The skin was incised with a No. 15 blade, and the skin and orbicularis muscle were divided using a high-frequency electrosurgical device. A traction suture was placed in the tarsus and pulled caudally. The orbital septum was opened, and the levator aponeurosis was exposed beneath the orbital fat.

**Figure 1:**
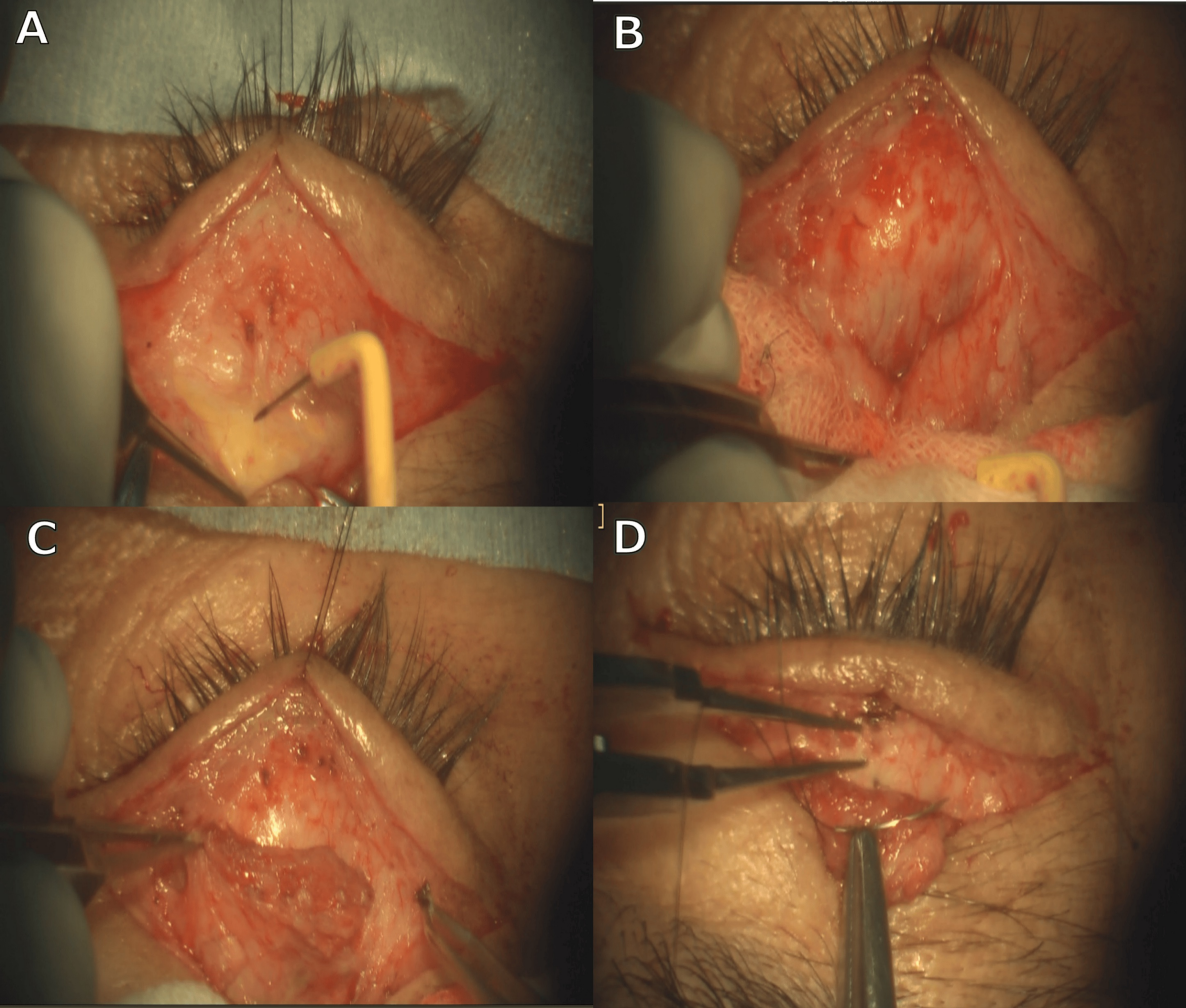
Surgical technique of transcutaneous levator aponeurosis recession. Intraoperative images from the surgeon's view. (A) The orbital septum is opened, and the levator aponeurosis is exposed beneath the orbital fat. (B) The levator aponeurosis is dissected from the anterior surface of the tarsus, and the Mueller muscle is detached from the upper tarsal border and conjunctival side. (C) The medial and lateral horns are divided, and the levator aponeurosis-Mueller muscle complex is released from the tarsus and conjunctiva. (D) The complex is recessed superiorly, and only the distal end of the levator aponeurosis is reattached to the tarsus with three 6-0 nylon sutures.

The levator aponeurosis was dissected from the anterior surface of the tarsus, and the Mueller muscle was detached from the upper tarsal border and the conjunctival side. The medial and lateral horns were divided, and the levator aponeurosis-Mueller muscle complex was released from the upper eyelid. The complex was recessed superiorly, and upper eyelid lengthening was confirmed intraoperatively. Only the distal end of the levator aponeurosis was reattached to the tarsus with three 6-0 nylon sutures. The Mueller muscle was left detached from the tarsus. The skin was closed with 6-0 nylon sutures. The surgical steps are illustrated in Figure 1.

Outcome summary

The primary outcome was the change in operated-eyelid UEMHR from baseline to approximately three months postoperatively. Secondary outcomes included operated-eyelid PFHR, intereye asymmetry in PFHR and UEMHR, exposure-related symptoms, and postoperative adverse events. The intereye difference in PFHR and UEMHR was calculated as the absolute difference between the affected and fellow eyes. Change was calculated as the postoperative value minus the preoperative value. Because the surgery was performed to correct upper eyelid retraction, decreases in operated-eyelid PFHR and UEMHR and reductions in intereye asymmetry were considered changes in the intended direction.

Because the number of patients was small, the analysis was descriptive and focused on individual paired changes. No inferential statistical testing was performed. Exposure-related keratopathy and lagophthalmos were assessed retrospectively from slit-lamp examination findings and medical records. Superficial punctate keratopathy (SPK) severity was recorded using the area-density grading system, in which the area of punctate epithelial staining is graded from A0 to A3 and the density is graded from D0 to D3 [12]. Postoperative adverse events assessed from the medical records included overcorrection, undercorrection, eyelid contour abnormality, diplopia, infection, hematoma, worsening lagophthalmos, and need for reoperation.

The study included five patients and five operated eyelids. Age ranged from 32 to 78 years. The etiologies were postoperative eyelid asymmetry after levator surgery in one case, cicatricial upper eyelid retraction after previous eyelid surgery in two cases, and TED-related or suspected inactive TED-related upper eyelid retraction in two cases. The clinical activity score (CAS), assessed according to the published system, was 0 in both TED-related and suspected TED-related cases [13]. The operated eyelid was the right upper eyelid in three cases and the left upper eyelid in two cases. Patient and operative characteristics are summarized in Table 1.

**Table 1:** Patient and operative characteristics CAS was 0 in Cases 4 and 5. In Case 2, postoperative image analysis used the final appearance after additional left upper eyelid levator advancement. "None" indicates that none of the prespecified adverse events occurred. Thyroid function and thyroid antibody data were not available for Case 4. TED: thyroid eye disease; CAS: clinical activity score; PFHR: palpebral fissure height ratio

Case	Age/sex	Etiology	Operated eyelid	Recession amount	Additional procedure	Evaluation	Adverse events
1	75/F	Postoperative asymmetry after bilateral levator surgery	Right	3 mm	None	Three months	None
2	70/M	Cicatricial retraction after previous eyelid surgery	Right	5 mm	Additional left levator advancement; final postoperative appearance analyzed	Three months	None
3	78/F	Cicatricial retraction after previous entropion surgery	Left	5 mm	Left upper eyelid skin excision	Three months	Lower PFHR than the fellow eye; no reoperation
4	32/F	TED-related left upper eyelid retraction	Left	5 mm	None	Three months	None
5	49/F	Suspected inactive TED-related right upper eyelid retraction	Right	5 mm	None	Three months	None

The median operated-eyelid PFHR decreased from 111.18% (range, 63.57-132.16%) preoperatively to 89.93% (range, 59.50-99.71%) postoperatively. The median change was -20.07 points (range, -51.35 to -4.07 points), and PFHR decreased in all five operated eyelids. The median operated-eyelid UEMHR decreased from 57.59% (range, 28.20-77.38%) to 37.72% (range, 25.17-50.09%). The median change was -23.31 points (range, -39.66 to -3.04 points), and UEMHR decreased in all five operated eyelids (Table 2).

**Table 2:** Preoperative and postoperative PFHR and UEMHR of the operated eyelid Values are the means of three measurements. Change was calculated as postoperative value minus preoperative value. PFHR: palpebral fissure height ratio; UEMHR: upper eyelid margin height ratio

Case	Operated eyelid	Preoperative PFHR (%)	Postoperative PFHR (%)	PFHR change (points)	Preoperative UEMHR (%)	Postoperative UEMHR (%)	UEMHR change (points)
1	Right	63.57	59.5	-4.07	28.2	25.17	-3.04
2	Right	111.18	99.71	-11.47	57.59	50.09	-7.49
3	Left	131.72	80.37	-51.35	77.38	37.72	-39.66
4	Left	110	89.93	-20.07	57.35	34.04	-23.31
5	Right	132.16	97.56	-34.6	76.99	43.54	-33.46

A lower postoperative PFHR indicates a smaller overall palpebral fissure height relative to WTW, whereas a lower postoperative UEMHR indicates an inferior shift of the upper eyelid margin relative to the corneal center.

Intereye asymmetry also decreased. The median PFHR asymmetry decreased from 20.97 points (range, 8.44-36.47 points) preoperatively to 3.54 points (range, 1.18-10.78 points) postoperatively. The median UEMHR asymmetry decreased from 21.89 points (range, 8.18-39.31 points) to 2.95 points (range, 0.54-17.49 points). Both PFHR asymmetry and UEMHR asymmetry decreased in all five cases (Table 3). Because Case 2 underwent additional contralateral levator advancement and Case 3 underwent concurrent skin excision, intereye asymmetry outcomes should be interpreted as secondary descriptive findings rather than isolated effects of operated-eyelid levator aponeurosis recession.

**Table 3:** Intereye asymmetry in PFHR and UEMHR Asymmetry was calculated as the absolute difference between the affected and fellow eyes. Change was calculated as postoperative asymmetry minus preoperative asymmetry. PFHR: palpebral fissure height ratio; UEMHR: upper eyelid margin height ratio

Case	Preoperative PFHR asymmetry (points)	Postoperative PFHR asymmetry (points)	PFHR asymmetry change (points)	Preoperative UEMHR asymmetry (points)	Postoperative UEMHR asymmetry (points)	UEMHR asymmetry change (points)
1	8.44	5.19	-3.25	8.18	6.19	-1.99
2	20.97	10.78	-10.19	18.77	17.49	-1.28
3	36.47	3.54	-32.93	30.5	2.14	-28.36
4	19.31	1.5	-17.81	21.89	0.54	-21.35
5	36.19	1.18	-35.02	39.31	2.95	-36.35

No patient required reoperation during the three-month follow-up. No infection, hematoma, diplopia, new severe lagophthalmos, or overcorrection/undercorrection requiring reoperation was observed. Preoperative exposure-related keratopathy was documented as SPK A1D1 in Case 2, SPK A2D1 in Case 3, and SPK A1D1 in Case 5; keratopathy was not observed postoperatively in any of these cases. In Case 4, approximately 2 mm of lagophthalmos was present preoperatively and resolved postoperatively. In Case 3, the postoperative PFHR of the operated eyelid was lower than that of the fellow eyelid; however, the marked preoperative upper eyelid retraction and corneal contact symptoms improved, there was no visual axis obstruction or functional complaint attributable to the postoperative eyelid position, and no additional surgery was requested or performed during follow-up. Eye-level image analysis values, including the mean and standard deviation of the three repeated measurements for each eye at each time point, are shown in Table 4. These standard deviations describe within-image repeatability by a single non-masked observer and do not represent formal measurement reliability.

**Table 4:** Eye-level image analysis values Values are shown as the mean (standard deviation) of three repeated measurements within each image by a single non-masked observer. These standard deviations reflect the within-image repeatability of triplicate measurements by one observer and should not be interpreted as evidence of intraobserver or interobserver measurement reliability. The patient, not each repeated measurement, was the independent statistical unit. PFHR: palpebral fissure height ratio; UEMHR: upper eyelid margin height ratio

Case	Time point	Eye	PFHR, mean (standard deviation) (%)	UEMHR, mean (standard deviation) (%)
1	Preoperative	Right	63.57 (0.33)	28.20 (0.10)
1	Preoperative	Left	55.13 (0.33)	20.02 (0.08)
1	Postoperative	Right	59.50 (0.29)	25.17 (0.17)
1	Postoperative	Left	54.32 (0.33)	18.97 (0.37)
2	Preoperative	Right	111.18 (0.75)	57.59 (0.29)
2	Preoperative	Left	90.21 (0.09)	38.82 (0.43)
2	Postoperative	Right	99.71 (0.10)	50.09 (0.10)
2	Postoperative	Left	88.93 (0.07)	32.61 (0.18)
3	Preoperative	Right	95.25 (0.15)	46.88 (0.10)
3	Preoperative	Left	131.72 (0.21)	77.38 (0.21)
3	Postoperative	Right	83.91 (0.11)	39.86 (0.16)
3	Postoperative	Left	80.37 (0.44)	37.72 (0.12)
4	Preoperative	Right	90.69 (0.03)	35.46 (0.25)
4	Preoperative	Left	110.00 (0.10)	57.35 (0.22)
4	Postoperative	Right	88.43 (0.41)	33.50 (0.36)
4	Postoperative	Left	89.93 (0.18)	34.04 (0.06)
5	Preoperative	Right	132.16 (0.20)	76.99 (0.11)
5	Preoperative	Left	95.97 (0.86)	37.69 (0.25)
5	Postoperative	Right	97.56 (0.50)	43.54 (0.78)
5	Postoperative	Left	98.74 (0.15)	40.58 (0.21)

Individual case descriptions

Case 1

**Figure 2:**
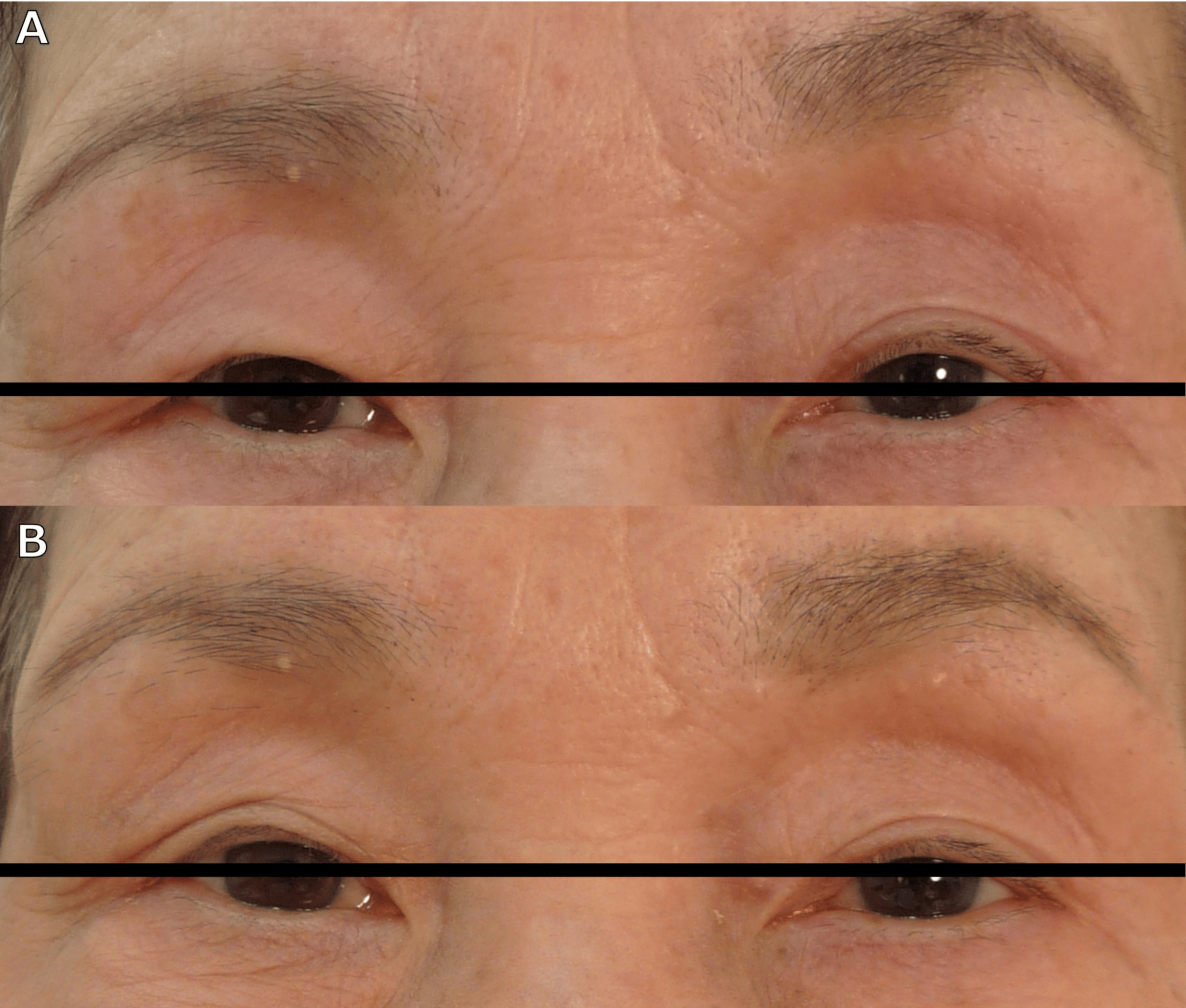
Case 1, a 75-year-old woman with postoperative upper eyelid asymmetry. (A) Preoperative appearance after bilateral levator surgery. PFHR was 63.57% in the right eye and 55.13% in the left eye; UEMHR was 28.20% in the right eye and 20.02% in the left eye. (B) Postoperative appearance after right upper eyelid levator aponeurosis recession. PFHR was 59.50% in the right eye and 54.32% in the left eye; UEMHR was 25.17% in the right eye and 18.97% in the left eye. PFHR: palpebral fissure height ratio; UEMHR: upper eyelid margin height ratio

A 75-year-old woman presented with intereye asymmetry in palpebral fissure height after bilateral levator surgery for asymmetric blepharoptosis. She felt that her right upper eyelid was excessively elevated and requested lowering of the right upper eyelid to match the left upper eyelid, which was lower. Preoperative PFHR was 63.57% in the right eye and 55.13% in the left eye; UEMHR was 28.20% in the right eye and 20.02% in the left eye. Right upper eyelid levator aponeurosis recession was performed with a recession amount of 3 mm. The operation was completed with the right eyelid margin positioned approximately 1 mm higher than the left intraoperatively. Postoperative PFHR was 59.50% in the right eye and 54.32% in the left eye; UEMHR was 25.17% in the right eye and 18.97% in the left eye. Eyebrow height and upper eyelid crease asymmetry improved clinically (Figure 2).

Case 2

**Figure 3:**
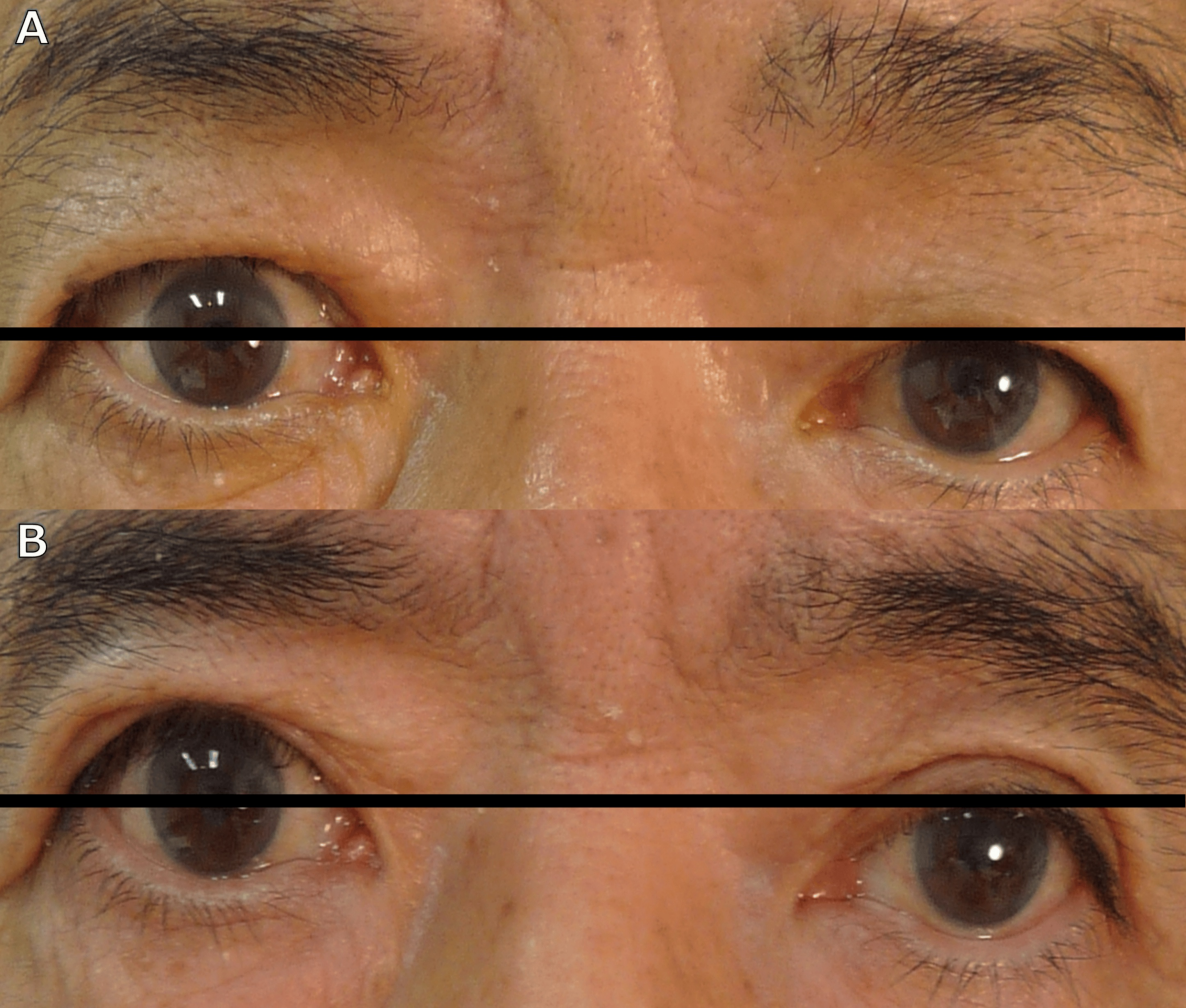
Case 2, a 70-year-old man with cicatricial right upper eyelid retraction after previous eyelid surgery. (A) Preoperative appearance. PFHR was 111.18% in the right eye and 90.21% in the left eye; UEMHR was 57.59% in the right eye and 38.82% in the left eye. (B) Final postoperative appearance after right levator aponeurosis recession and additional left levator advancement. PFHR was 99.71% in the right eye and 88.93% in the left eye; UEMHR was 50.09% in the right eye and 32.61% in the left eye. PFHR: palpebral fissure height ratio; UEMHR: upper eyelid margin height ratio

A 70-year-old man was referred for right upper eyelid retraction, intereye asymmetry, lagophthalmos, foreign body sensation, and punctate epithelial keratopathy documented as SPK A1D1 after previous right upper eyelid entropion surgery at another hospital. Preoperative PFHR was 111.18% in the right eye and 90.21% in the left eye; UEMHR was 57.59% in the right eye and 38.82% in the left eye. Because exposure-related keratopathy was present, right upper eyelid levator aponeurosis recession was performed. Severe cicatricial changes were encountered, and the levator aponeurosis and Mueller muscle were dissected extensively. The eyelid margin was lowered intraoperatively to slightly below the superior corneal limbus. Additional left upper eyelid levator advancement was subsequently performed after the contralateral upper eyelid position changed in a manner consistent with Hering's law [14]. At the final postoperative assessment, PFHR was 99.71% in the right eye and 88.93% in the left eye; UEMHR was 50.09% in the right eye and 32.61% in the left eye. Postoperative keratopathy was not observed (Figure 3).

Case 3

**Figure 4:**
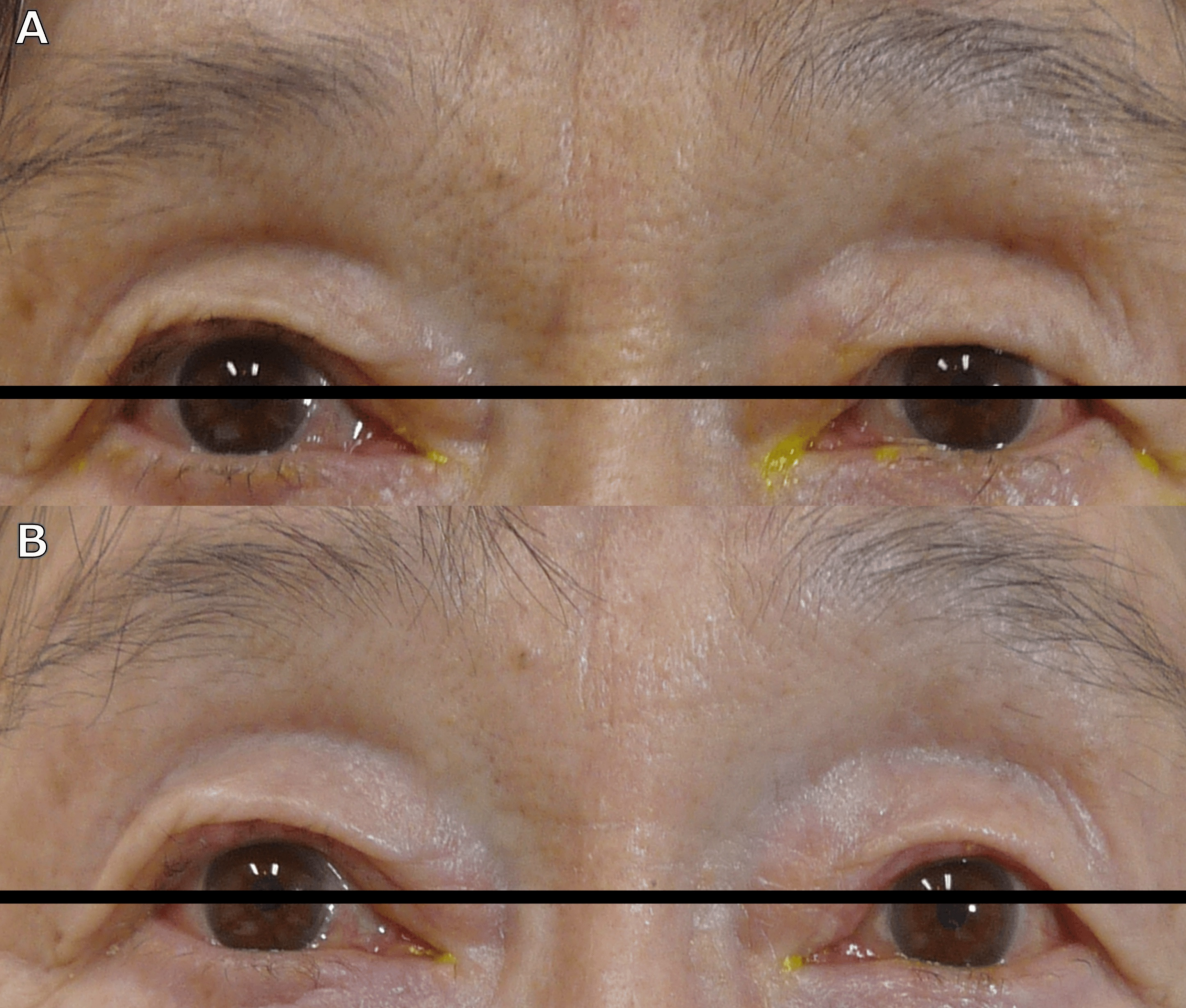
Case 3, a 78-year-old woman with cicatricial left upper eyelid retraction after previous entropion surgery. (A) Preoperative appearance. PFHR was 95.25% in the right eye and 131.72% in the left eye; UEMHR was 46.88% in the right eye and 77.38% in the left eye. (B) Postoperative appearance after left levator aponeurosis recession and left upper eyelid skin excision. PFHR was 83.91% in the right eye and 80.37% in the left eye; UEMHR was 39.86% in the right eye and 37.72% in the left eye. PFHR: palpebral fissure height ratio; UEMHR: upper eyelid margin height ratio

A 78-year-old woman presented with left eye foreign body sensation and conjunctival injection after previous suture-based surgery for left upper eyelid entropion. Left upper eyelid retraction was present, and redundant anterior lamellar tissue crossed the eyelid margin and contacted the cornea, causing punctate epithelial keratopathy documented as SPK A2D1. Preoperative PFHR was 95.25% in the right eye and 131.72% in the left eye; UEMHR was 46.88% in the right eye and 77.38% in the left eye. Left upper eyelid levator aponeurosis recession and left upper eyelid skin excision were performed. Severe cicatricial changes were present; therefore, the levator aponeurosis and Mueller muscle were carefully dissected. Intraoperatively, the eyelid margin was lowered to approximately 1 mm below the superior corneal limbus. Postoperative PFHR was 83.91% in the right eye and 80.37% in the left eye; UEMHR was 39.86% in the right eye and 37.72% in the left eye. Although the operated eyelid showed a lower PFHR than the fellow eyelid postoperatively, corneal contact symptoms improved, postoperative keratopathy was not observed, there was no visual axis obstruction or functional complaint, and the eyelid position was considered clinically acceptable (Figure 4).

Case 4

A 32-year-old woman was referred for left upper eyelid retraction and foreign body sensation related to TED. She had previously received two local triamcinolone injections of 10 mg between the conjunctival side and the Mueller muscle region at another clinic because mild inflammatory findings had been observed on magnetic resonance imaging. Eyelid swelling improved, and no change was observed during two years of follow-up. The CAS was 0. Preoperative magnetic resonance imaging findings were reviewed during endocrinology consultation and were considered not to indicate active orbital inflammation. Thyroid function and thyroid antibody laboratory values were not available in the medical record; therefore, reference ranges and units could not be reported for this case. Approximately 2 mm of lagophthalmos persisted. Preoperative PFHR was 90.69% in the right eye and 110.00% in the left eye; UEMHR was 35.46% in the right eye and 57.35% in the left eye. Left upper eyelid levator aponeurosis recession was performed through a skin incision 7 mm above the eyelid margin. The levator aponeurosis was recessed 5 mm from its tarsal attachment. Lagophthalmos resolved postoperatively. Postoperative PFHR was 88.43% in the right eye and 89.93% in the left eye; UEMHR was 33.50% in the right eye and 34.04% in the left eye. No clinical images are presented for this case.

Case 5

**Figure 5:**
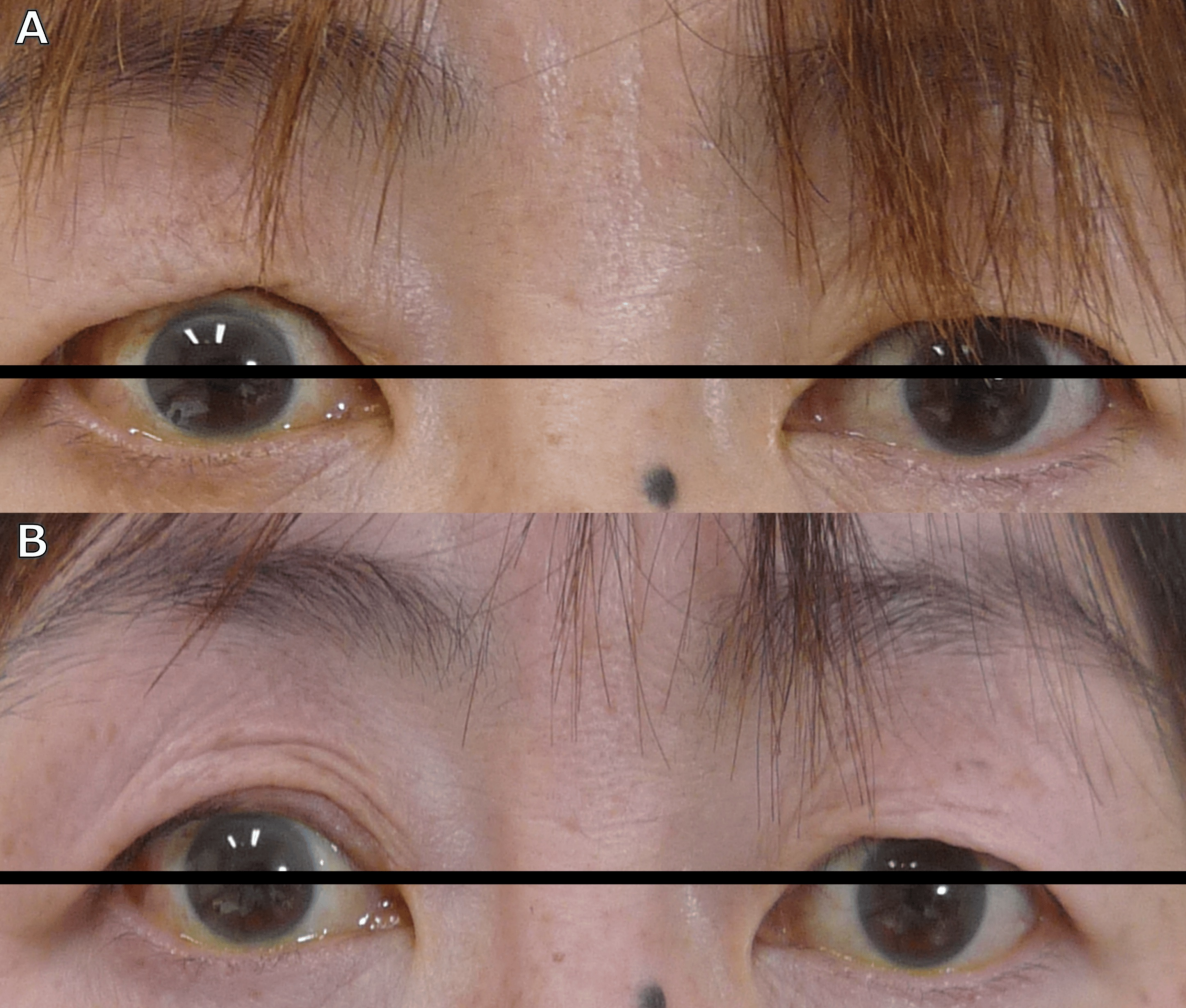
Case 5, a 49-year-old woman with suspected inactive thyroid eye disease-related right upper eyelid retraction and right upper eyelid entropion. (A) Preoperative appearance. PFHR was 132.16% in the right eye and 95.97% in the left eye; UEMHR was 76.99% in the right eye and 37.69% in the left eye. (B) Postoperative appearance after right upper eyelid levator aponeurosis recession. PFHR was 97.56% in the right eye and 98.74% in the left eye; UEMHR was 43.54% in the right eye and 40.58% in the left eye. PFHR: palpebral fissure height ratio; UEMHR: upper eyelid margin height ratio

A 49-year-old woman was referred for right upper eyelid retraction, right upper eyelid entropion, foreign body sensation, and punctate epithelial keratopathy documented as SPK A1D1. Similar findings had been present on eyelid images obtained more than one year earlier, and suspected inactive TED was considered. The CAS was 0. Orbital magnetic resonance imaging obtained after endocrinology consultation showed no obvious inflammation or enlargement of the levator muscle and no marked intereye difference in eyelid swelling. Thyroid function testing showed a thyroid-stimulating hormone (TSH) level of 2.79 µIU/mL (reference range, 0.61-4.23 µIU/mL), a free thyroxine (FT4) level of 0.68 ng/dL (reference range, 0.76-1.65 ng/dL), and a thyroid-stimulating antibody (TSAb) level of 107% (reference range, 0-110%). Although FT4 was slightly below the institutional reference range, there was no biochemical evidence of hyperthyroidism. Preoperative PFHR was 132.16% in the right eye and 95.97% in the left eye; UEMHR was 76.99% in the right eye and 37.69% in the left eye. Right upper eyelid levator aponeurosis recession was performed through a skin incision 5 mm above the eyelid margin. The levator aponeurosis was recessed 5 mm from its tarsal attachment. The intraoperative target was to leave the right eyelid margin approximately 1 mm higher than the left. Postoperative keratopathy resolved. Postoperative PFHR was 97.56% in the right eye and 98.74% in the left eye; UEMHR was 43.54% in the right eye and 40.58% in the left eye (Figure 5).

## Discussion

This case series describes a transcutaneous levator aponeurosis recession technique that does not require spacer grafts or newly created flaps. Previously reported surgical options for upper eyelid retraction include full-thickness anterior blepharotomy [3], modified marginal myotomy [4], adjustable levator recession [5], orbital septal flap-based levator lengthening [6-8], V-Y levator lengthening [9], and stepwise strategies for iatrogenic retraction [10]. These procedures have reported utility; however, each requires technique-specific maneuvers such as full-thickness eyelid incision, adjustable suturing, flap or spacer preparation, or V-Y plasty [3-10].

The present technique differs from orbital septal flap-based procedures [6-8] in that it uses neither a spacer graft nor a newly created flap. It shares the initial transcutaneous exposure of the levator aponeurosis with common ptosis surgery; however, its purpose and dissection are different. To lengthen the upper eyelid, the levator aponeurosis is dissected from the anterior tarsal surface, the Mueller muscle is widely detached from the upper tarsal border and conjunctival side, the medial and lateral horns are divided, and the levator aponeurosis-Mueller muscle complex is recessed. Thus, the dissection is broader than in typical involutional ptosis surgery. The practical feature of this method is that upper eyelid lengthening is achieved by reattaching only the distal end of the levator aponeurosis to the tarsus without harvesting tissue or securing a spacer. However, in severe cicatricial cases, such as Cases 2 and 3, dissection can be demanding and should not be considered a simple procedure.

The present findings support the feasibility of performing upper eyelid lowering through a transcutaneous approach without spacer grafts or newly created flaps in carefully selected cases. In all five operated eyelids, both WTW-normalized indices, PFHR and UEMHR, decreased postoperatively, suggesting consistent eyelid lowering despite heterogeneous etiologies. The technique may be particularly suitable for selected patients with stable upper eyelid retraction, including inactive TED-related retraction or postoperative/cicatricial retraction in which adequate release of the levator aponeurosis-Mueller muscle complex can be achieved. However, because this series was small and follow-up was limited to three months, longer-term data are needed to determine durability, recurrence, late contour changes, and etiology-specific indications.

In this series, both operated-eyelid PFHR and UEMHR decreased in all five cases. PFHR is useful for summarizing the overall palpebral fissure height but is affected by both upper and lower eyelid positions. UEMHR, defined as the WTW-normalized distance from the corneal center to the upper eyelid margin, is a photographic index that more directly reflects upper eyelid margin position. A lower postoperative PFHR indicates a smaller overall palpebral fissure height relative to WTW, whereas a lower postoperative UEMHR indicates an inferior shift of the upper eyelid margin relative to the corneal center. The consistent decrease in UEMHR suggests that the surgery lowered the upper eyelid margin itself, not only the total palpebral fissure height. These indices were used only for within-series descriptive comparison.

The etiologies in this series were heterogeneous. Case 1 was a revision case after levator surgery with relatively little scarring, and the postoperative eyelid position was close to the intraoperative target. Cases 2 and 3 had cicatricial retraction after previous eyelid surgery, and extensive release of scar tissue around the levator aponeurosis-Mueller muscle complex was required. These cases may have a risk of postoperative recurrent cicatricial change or regression. In addition, Case 2 underwent additional contralateral levator advancement, and Case 3 underwent concurrent skin excision. The additional contralateral procedure in Case 2 was consistent with the recognized risk of contralateral eyelid position change after unilateral eyelid surgery [14]. Accordingly, operated-eyelid PFHR and UEMHR changes provide the most direct descriptive evidence of eyelid lowering in this series, whereas intereye asymmetry outcomes should be interpreted as secondary descriptive findings because intereye asymmetry in Case 2 was influenced by the additional contralateral procedure, and intereye asymmetry in Case 3 should be interpreted with the concurrent skin excision in mind.

Cases 4 and 5 involved TED-related or suspected inactive TED-related upper eyelid retraction without previous eyelid surgery. In these patients, dissection was relatively easier than in the cicatricial cases. The CAS was 0 in both cases. In Case 4, preoperative magnetic resonance imaging findings were reviewed during endocrinology consultation and were considered not to indicate active orbital inflammation; however, thyroid function and thyroid antibody data were not available in the medical record. In Case 5, orbital magnetic resonance imaging showed no obvious levator inflammation or enlargement, TSAb was within the institutional reference range, and there was no biochemical evidence of hyperthyroidism, although FT4 was slightly below the institutional reference range. Rehabilitative eyelid surgery in TED should generally be considered after inflammatory activity and thyroid status have stabilized [2]. The present series is too small to define etiology-specific target eyelid positions or recession amounts; however, it suggests that tissue characteristics, cicatricial change, and TED-related changes in the Mueller muscle or levator complex may influence postoperative eyelid position. Previous studies of ptosis surgery have shown that intraoperative eyelid position is related to postoperative eyelid position, although postoperative prediction remains imperfect and multifactorial [15,16]. Matsuo reported that stretching of the Mueller muscle may induce involuntary contraction of the levator muscle, supporting a functional relationship between the Mueller muscle and levator activity [17]. However, the mechanisms of postoperative eyelid position change after levator recession, especially in TED, cannot be determined from this small series.

A central methodological constraint of this study is that PFHR and UEMHR are non-standard, unvalidated photographic indices rather than established clinical measurements. Margin reflex distance 1 (MRD1) is the standard clinical metric for upper eyelid position and retraction [18]. MRD1 was not measured prospectively in this retrospective series. Although a corneal light reflex was visible in some photographs, the images were not obtained with a millimeter scale; therefore, retrospective MRD1 values would have required WTW-based calibration and would have remained dependent on the same photographic assumptions that limit PFHR and UEMHR. WTW-based normalization may be affected by gaze, head position, camera angle, corneal visibility, and image selection. Consequently, PFHR and UEMHR should be interpreted only as within-series descriptive photographic indices and should not be considered substitutes for prospectively measured MRD1.

This study has several limitations. First, it included only five patients, and the etiologies were heterogeneous. Second, this was a retrospective case series without a control group. Third, follow-up was limited to three months, so long-term stability, recurrence, and etiology-specific durability could not be assessed. Fourth, all image measurements were performed by a single non-masked observer, which may have introduced measurement bias. The triplicate-measurement standard deviations in Table 4 reflect only within-image repeatability by one observer and should not be interpreted as formal intraobserver or interobserver reliability; no intraclass correlation coefficient was assessed. Fifth, the absence of prospectively measured MRD1 is the central methodological limitation of this study. PFHR and UEMHR were derived from photographs and normalized to WTW; these are non-standard, unvalidated photographic indices without established normative values. WTW-based normalization may be affected by gaze, head position, camera angle, corneal visibility, and image selection, and therefore limits comparability with studies that report MRD1. Sixth, PFHR is affected by both upper and lower eyelid positions, whereas UEMHR is a relative photographic index rather than a direct clinical measurement of upper eyelid position. Seventh, although preoperative SPK was graded using the area-density system when documented [12], comprehensive standardized ocular surface scoring was not available for all patients. Eighth, complete thyroid function and thyroid antibody data were not available for Case 4, and TED activity assessment relied on clinical stability, CAS, magnetic resonance imaging, and endocrinology assessment, where available. Future studies should include larger cohorts, longer follow-up, standardized ocular surface assessment, prospective MRD1 measurement, masked independent graders, and formal measurement reproducibility analysis.

## Conclusions

Transcutaneous levator aponeurosis recession without spacer grafts or newly created flaps was feasible in these selected cases and was associated with consistent early lowering of the upper eyelid when assessed using WTW-normalized photographic eyelid height indices. These findings should be interpreted as preliminary feasibility data rather than evidence of established clinical efficacy because the series was small and heterogeneous, follow-up was limited to three months, measurements were performed by a single non-masked observer without formal reliability testing, and MRD1 was not measured prospectively. Further prospective studies with larger cohorts, standardized MRD1 and ocular surface measurements, masked independent graders, formal reproducibility analysis, and longer-term follow-up are needed to determine durability, recurrence, late eyelid contour changes, and etiology-specific suitability.
